# Genome-Wide Identification and Characterization of Ammonium Transporter (AMT) Genes in *Chlamydomonas reinhardtii*

**DOI:** 10.3390/genes15081002

**Published:** 2024-07-31

**Authors:** Wenhui Hu, Dan Wang, Shuangshuang Zhao, Jiaqi Ji, Jing Yang, Yiqin Wan, Chao Yu

**Affiliations:** 1 School of Life Sciences, Nanchang University, Nanchang 330031, China; 405600220102@email.ncu.edu.cn (W.H.); 405600230110@email.ncu.edu.cn (D.W.); zss09163@163.com (S.Z.); 5601123018@email.ncu.edu.cn (J.J.); yangjing@ncu.edu.cn (J.Y.); 2 Basic Experimental Center of Biology, Nanchang University, Nanchang 330031, China

**Keywords:** *Chlamydomonas reinhardti*, CrAMTs, ammonium transporters, bioinformatics, AMT1 family

## Abstract

Ammonium transporters (AMTs) are vital plasma membrane proteins facilitating NH_4_^+^ uptake and transport, crucial for plant growth. The identification of favorable AMT genes is the main goal of improving ammonium-tolerant algas. However, there have been no reports on the systematic identification and expression analysis of *Chlamydomonas reinhardtii* (*C. reinhardtii*) AMT genes. This study comprehensively identified eight CrAMT genes, distributed across eight chromosomes, all containing more than 10 transmembrane structures. Phylogenetic analysis revealed that all CrAMTs belonged to the AMT1 subfamily. The conserved motifs and domains of CrAMTs were similar to those of the AMT1 members of OsAMTs and AtAMTs. Notably, the gene fragments of CrAMTs are longer and contain more introns compared to those of AtAMTs and OsAMTs. And the promoter regions of CrAMTs are enriched with *cis*-elements associated with plant hormones and light response. Under NH_4_^+^ treatment, *CrAMT1;1* and *CrAMT1;3* were significantly upregulated, while *CrAMT1;2*, *CrAMT1;4*, and *CrAMT1;6* saw a notable decrease. *CrAMT1;7* and *CrAMT1;8* also experienced a decline, albeit less pronounced. Transgenic algas with overexpressed *CrAMT1;7* did not show a significant difference in growth compared to CC-125, while transgenic algas with *CrAMT1;7* knockdown exhibited growth inhibition. Transgenic algas with overexpressed or knocked-down *CrAMT1;8* displayed reduced growth compared to CC-125, which also resulted in the suppression of other CrAMT genes. None of the transgenic algas showed better growth than CC-125 at high ammonium levels. In summary, our study has unveiled the potential role of CrAMT genes in high-ammonium environments and can serve as a foundational research platform for investigating ammonium-tolerant algal species.

## 1. Introduction

Nitrogen, a crucial component of amino acids and nucleic acids, is essential for plant growth and development. Soil contains ammonium and nitrate nitrogen, which are available to plants, and nitrate and ammonium nitrogen are considered the main sources of nitrogen for plants. In flooded soils, rich in or containing both ammonium and nitrate, plants prefer ammonium as their nitrogen source due to its lower energy requirement for uptake and assimilation compared to nitrate [1,2,3], especially in nitrogen-deficient plants [4,5]. Ammonium, the primary inorganic nitrogen source, is transported across membranes primarily through ammonium transporters (AMTs) [6,7], which are important carriers for plant absorption and utilization of ammonium, balancing the concentration of ammonium in plants by regulating the absorption of ammonium in the environment and the transport of ammonium in various organs and tissues of plants [8,9,10]. Kinetic [11,12,13] experiments indicate that plant AMTs can be classified into high-affinity (HATSs) and low-affinity (LATSs) ammonium transporters, which closely and dynamically regulate ammonium uptake during plant growth and development. When the ammonium concentration is in the sub-millimolar range, plants primarily use HATSs to absorb ammonium, and when the available ammonium concentration is in the millimolar range, LATSs are utilized [14,15].

AMTs are essential for the cellular uptake of ammonium and are found across a wide range of species [16,17,18], exhibiting high affinity for NH_4_^+^ in yeast, bacteria, and mammals [19,20]. The first plant AMT was identified in *Arabidopsis thaliana*, demonstrating its ability to compensate for ammonium absorption deficiencies in yeast mutants [21]. In *Arabidopsis*, six AMT genes have been identified, with *AtAMT1;1* mRNA expression levels closely correlated with plant nitrogen status changes [22]. *AtAMT1;1* and *AtAMT1;3* account for 30–35% of NH_4_^+^ uptake in nitrogen-deficient roots [21], while *AtAMT1;2* contributes 18–26% [16]. Research on rice showed that the knockdown of *OsAMT1;1*, *OsAMT1;2*, and *OsAMT1;3* led to a 95% reduction in ammonium uptake by roots, highlighting the necessity of these AMT1 members for ammonium uptake and their varied regulatory mechanisms in response to ammonium levels [23]. The AMT gene family is categorized into subfamilies based on amino acid sequences, notably the AMT1 and AMT2 subfamilies [23,24,25], and the subfamily gene members have different contributions to ammonium uptake. AMT1 proteins primarily facilitate NH_4_^+^ transport, whereas AMT2 and AMT1 proteins are related to the methylamine permease (MEP) family [25], despite AMT2 proteins having markedly different primary structures from AMT1 proteins but similar higher-order structures [10]. Compared with the AMT1 subfamily, there are relatively few reports on the AMT2 subfamily, but related reports prove that the AMT2 subfamily also acts on the uptake and transport of ammonium in plants. For instance, *AtAMT2;1* mediates the accumulation of ammonium in xylem fluid [26], and in sugarcane, *ScAMT2;1* enhances root-to-stem ammonium transport under high-NH_4_^+^ conditions [27]. Additionally, in sugarcane, *ScAMT3;3* expressed in young and mature leaves acts in NH_4_^+^ trafficking as a low-affinity transporter [27]. In cassava, MeAMT2.3, MeAMT2.5, and MeAMT2.6 respond to ammonium deficiency [28]. CsAMT2.2 and CsAMT2.3 are highly expressed in the roots, suggesting that they play a major role in controlling the uptake of NH_4_^+^ by the roots [29]. Rice possesses at least 10 AMT2 homologs, categorized into AMT2, AMT3, AMT4, and AMT5 clusters within the AMT2 subfamily [30,31,32,33]. In rice some AMT2 transporters are expressed differently in different varieties with different nitrogen requirements [34,35].

Excessive NH_4_^+^ concentrations, however, are toxic, inhibiting plant growth, disrupting photosynthesis, and causing plant yellowing [36,37,38,39,40]. Therefore, regulating the concentration of NH_4_^+^ in plants is essential for the healthy growth of plants. High concentrations of ammonium nitrogen typically lead to toxic effects in plants associated with ion, metabolism, and hormone imbalances [4,41]. For instance, ammonium-sensitive plants such as citrus, wheat, and rice are usually inhibited in terms of growth when exposed to excessive levels of ammonium nitrogen [42,43,44,45]. This inhibition is characterized by shortened primary roots, decreased root vitality, leaf chlorosis and wilting, suppressed seedling growth, and reduced biomass accumulation [36,46,47]. By studying the sensitivity of 19 common algal species in different nutrient types of water bodies to ammonium toxicity, it has been revealed that elevated NH_4_^+^ levels in water bodies can also have toxic effects on algae [48]. In general, the causes of ammonium toxicity can be attributed to several factors: rhizosphere acidification, protein glycosylation defects, ineffective NH_4_^+^ cycling, the accumulation of reactive oxygen species (ROS), cytoplasm acidification, the depletion of inorganic cations and organic acids, damage to the photosystem, and the disruption of hormone signal transduction [40,49,50]. Therefore, to mitigate the toxic effects of NH_4_^+^ on plants, strategies such as clearing the accumulation of ammonium-dependent ROS in plant cells, storing NH_4_^+^ in vacuoles, or inhibiting NH_4_^+^ transport can be employed to alleviate ammonium toxicity [48]. For example, in rice, silencing *OsAMT1;1*, *OsAMT1;2*, and *OsAMT1;3* leads to a 95% reduction in ammonium uptake by the roots [23]. The overexpression of *LjAMT2;2* increases *Lotus japonicus*’ absorption of ammonium nitrogen, resulting in the doubling of nitrogen content in leaves and roots, thereby alleviating nitrogen stress and promoting plant growth [51]. While studies have shown that high ammonium levels can inhibit algal growth and initial research has been conducted on the molecular mechanisms of its toxicity [49], there is currently no research on the molecular mechanisms of ammonium root transport protein response to ammonium toxicity. Investigating the molecular mechanisms of ammonium root transport protein response to ammonium toxicity can serve as a direction for alleviating algal ammonium toxicity.

*C. reinhardtii*, a unicellular eukaryotic green alga, belongs to the Chlorophyta class within the Chlamydomonas family. This spherical or ovate algae is characterized by two equally long flagella at its anterior. Notable for its rapid reproduction, its ease of cultivation, and the presence of chloroplasts for photosynthesis, *C. reinhardtii* exhibits both plant-like and microbial traits. As a model organism, it boasts a well-defined genetic background and is distinguished as the sole biomaterial equipped with genetic transformation systems for the nucleus, chloroplast, and mitochondria, establishing it as a pivotal model species [52]. Nitrogen, a critical nutrient for *C. reinhardtii*, influences oil accumulation and the expression of various genes related to nitrogen uptake and assimilation under deficient conditions [53]. *C. reinhardtii* prefers ammonium (NH_4_^+^) as a nitrogen source, but its late-stage growth under high-NH_4_^+^ concentrations (0.5 ∼ 1 g/L) is retarded due to medium acidification [54]. Ammonium transporters play a crucial role in its nitrogen assimilation pathway, facilitating the uptake of ammonium ions for nutrient acquisition [55]. Although we speculate that high ammonium levels can cause the inhibition of *C. reinhardtii* growth, which may be related to ammonium transporters, there is currently limited research on its ammonium transporters. Moreover, the identification of favorable AMT genes can be used in the cultivation of ammonium-tolerant algal species. This study systematically analyzes the ammonium transporter proteins of *C. reinhardtii*, identifying the AMT gene and thoroughly examining its localization, structure, promoter, and conserved motifs. We compared the growth and gene expression profiles of transgenic algas with overexpressed and knocked-down *CrAMT1;7* and *CrAMT1;8* to understand the specific effects of these gene manipulations. Simultaneously, to investigate whether the transgenic algas possess the ability to tolerate high levels of ammonium, we exposed the transgenic algas to high levels of ammonium toxicity to assess the potential contributions of these genes in alleviating ammonium toxicity. These analyses provide a theoretical foundation for understanding the function of genes within *C. reinhardtii* AMTs. This study aims to delve deeper into the function and regulatory mechanisms of AMTs in regulating ammonium uptake and metabolism in *C. reinhardtii*, it also provides a mitigation plan and research basis for ammonium toxicity on algae.

## 2. Materials and Methods

### 2.1. Identification and Bioinformatics Analyses of CrAMTs

Genomic data and annotations for *C. reinhardtii* (Chlamydomonas reinhardtii v5.6), *Arabidopsis* (Arabidopsis thaliana TAIR10), rice(Oryza sativa v7.0), and *Physcomitrium patens* (Physcomitrium patens v3.3) were retrieved from the Phytozome database (https://phytozome-next.jgi.doe.gov/, accessed on 21 October 2023), and for *Chlorella vulgaris* from the National Center for Biotechnology Information (https://www.ncbi.nlm.nih.gov/, accessed on 21 October 2023). Utilizing the conserved domains of AMT proteins in *Arabidopsis* and rice as references, Blastp analysis was performed to identify potential *C. reinhardtii* AMT genes. These candidates were further refined through BLAST searches and HMMER v3.1 [56] analysis, with a threshold set at 0.001. The identified AMT genes in *C. reinhardtii* were named following the nomenclature established by David González-Ballester et al. [57]. Subcellular localization of the CrAMT genes was predicted using the WOLF-PSORT tool (https://wolfpsort.hgc.jp/, accessed on 23 October 2023) and TMHMM SERVER v2.0 (https://services.healthtech.dtu.dk/services/TMHMM-2.0/, accessed on 23 October 2023) was employed for predicting transmembrane domains of CrAMTs.

### 2.2. Phylogenetic Tree Analysis of CrAMTs

The full-length amino acid sequences of AMTs were sourced from the Phytozome and NCBI databases. These sequences were aligned using MEGA 11 and a phylogenetic tree was constructed employing the maximum-likelihood (ML) method, with bootstrap analysis performed on 1000 replicates. The evolutionary tree was visualized using the Evolview web-based tool (https://www.evolgenius.info/evolview/, accessed on 27 October 2023).

### 2.3. Gene Structures, Conserved Motifs, and Conserved Protein Domain Analyses of CrAMTs

AMT promoter sequences, CDSs, genomic sequences, and amino acid sequences were retrieved from the Phytozome database. Gene Structure Display Server 2.0 (http://gsds.gao-lab.org/, accessed on 24 October 2023) was utilized for analyzing AMT gene structures. Conserved motifs in AMT amino acid sequences were identified using the MEME v5.5.3 (https://meme- suite.org/meme/tools/meme, accessed on 24 October 2023), setting the number of motifs to 8. NCBI CD-search [58] (https://www.ncbi.nlm.nih.gov/Structure/cdd/wrpsb.cgi, accessed on 24 October 2023) was applied to analyze the conserved domains of CrAMTs. TBtools v2.030 [59] software integrated the phylogenetic tree, gene structures, conserved motifs, and domains.

### 2.4. Chromosome Localization Analysis and Collinearity Analysis of CrAMTs

Chromosomal location and gene structure information for CrAMTs were obtained from the GFF3 file and visualized using TBtools v2.030 [59,60]. This software was also employed to analyze the *C. reinhardtii* genome, extract collinearity gene information, and visualize it using the Advanced Circos function.

### 2.5. Cis-Element Analysis of CrAMT Promoter Regions

*Cis*-elements in the promoter regions of CrAMT genes were analyzed using TBtools v2.030 and the PlantCARE website, extracting a 2000 bp sequence upstream of the start codon for visual analysis. The MEME v5.5.4 (https://meme-suite.org/meme/tools/meme, accessed on 24 October 2023) was utilized to identify conserved motifs in the promoter regions of AMT genes from *Arabidopsis*, rice and *C. reinhardtii*, with the number of motifs set to 10.

### 2.6. Expression Patterns of CrAMT Genes under High-Ammonium Conditions

*C. reinhardtii* (CC-125) was grown in Tris–acetate–phosphate (TAP) medium supplemented with varying concentrations of ammonium (7 mM, 17.5 mM, 35 mM, and 70 mM NH_4_^+^) for three days, corresponding to the logarithmic growth phase. The expression levels of CrAMT genes were assessed through quantitative real-time PCR (qRT-PCR) after 48 and 72 h of ammonium treatment. Additionally, the daily optical density of CC-125 at 630 nm was measured to monitor growth.

RNA was isolated using the Trizol method from each sample. Subsequently, first-strand cDNA synthesis was performed using the HiScript^®^ III 1st Strand cDNA Synthesis Kit (+gDNA wiper). Specific primers for CrAMT genes (Table 1) were designed using Premier 5 software. The qRT-PCR reactions employed SYBR Green Realtime PCR Master Mix and were conducted on a TianLong GENTIER96 real-time PCR system. Expression levels of CrAMT genes, normalized to the Actin gene, were calculated using the 2^−ΔΔCt^ method [61].

Data visualization, including line and histogram plots of CC-125 optical density at 630 nm and CrAMT gene expression levels, was carried out using GraphPad Prism 10.

### 2.7. Construction and Phenotypic Characterization of Transgenic Algas

The forward and reverse sequences of *CrAMT1;7* and *CrAMT1;8* CDS were linked using the 35S promoter and then integrated into the pCAMBIA2300 vector, which carries the G418 resistance gene. These recombinant vectors were introduced into the wild-type CC-125 via Agrobacterium-mediated transformation. Transgenic algas were selected on solid TAP medium supplemented with 3 mg/mL G418, where algae carrying the G418 resistance gene could grow and survive, and designated as *CrAMT1;7* overexpression (CrAMT1;7-OE), *CrAMT1;7* knockdown (CrAMT1;7-KD), *CrAMT1;8* overexpression (CrAMT1;8-OE), and *CrAMT1;8* knockdown (CrAMT1;8-KD). From each group, 2–10 transgenic algas were analyzed. RNA was extracted, and *CrAMT1;7* and *CrAMT1;8′*s expression was verified by qRT-PCR. Expression levels of the remaining 7 CrAMT genes in the transgenic algas were compared with CC-125. The growth of the transgenic algas and CC-125 was evaluated in TAP medium, measuring the optical density at 680 nm, fresh weight per volume, and chlorophyll content per mass unit. We performed data visualization using GraphPad Prism 10.

### 2.8. Growth Trend of Transgenic algas under High-Ammonium Treatment

Treating CrAMT1;7-OE, CrAMT1;7-KD, CrAMT1;8-OE, and CrAMT1;8-KD with 35 mM NH_4_^+^ as toxic conditions, the absorbance values of the algas at 680 nm were recorded every 24 h. We performed data visualization using GraphPad Prism 10.

### 2.9. Statistical Analysis

All data were visualized in GraphPad Prism 10. The statistical analyses were performed using IBM SPSS Statistics v25.0.0. The significance among multiple groups was calculated using one-way ANOVA followed by Tukey’s test at *p* < 0.05 (*p* < 0.05 indicated by *, *p* < 0.01 indicated by **, *p* < 0.001 indicated by ***).

## 3. Results

### 3.1. Identification of CrAMTs and the Basic Information of CrAMTs

Using Blastp and HMMERv3.1, we identified eight AMT genes in the *C. reinhardtii* genome, corroborating the findings of David González-Ballester et al. [57]. These genes were named *CrAMT1;1* through *CrAMT1;8*, and the encoded proteins were designated as CrAMT1.1 to CrAMT1.8 (Table 2). The encoded proteins varied in length from 478 to 782 amino acids, with corresponding coding sequences ranging from 1446 bp to 2349 bp. Each protein contained between 10 and 11 transmembrane regions, indicating their localization to the plasma and vacuolar membranes. This suggests the CrAMTs’ dual role in regulating NH_4_^+^ transport across cellular membranes and storing NH_4_^+^ within vacuoles.

### 3.2. Phylogenetic Analyses of CrAMT Genes

**Figure 1:**
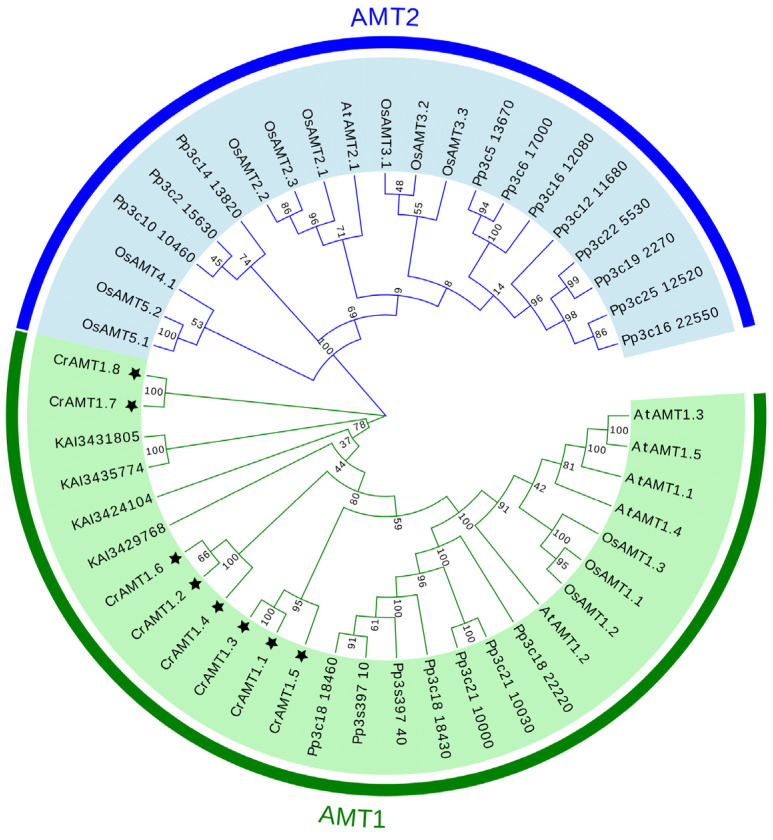
A phylogenetic tree of AMT family proteins. The phylogenetic tree was generated using MEGA 11, employing the neighbor-joining method with 1000 bootstrap replicates. The AMT proteins of the five species (CrAMT stands for *C. reinhardtii*, AtAMT stands for *Arabidopsis*, OsAMT stands for rice, Pp stands for *P. patens*, and KAI stands for *C. vulgaris*) were split into two clades, the AMT1 subfamily and the AMT2 subfamily. The figures in green denote the AMT1 subfamily, the figures in blue indicate the AMT2 subfamily, and the black stars highlight CrAMT proteins.

The evolutionary relationships among AMT genes were elucidated through the construction of a phylogenetic tree using the neighbor-joining method in MEGA 11. This analysis incorporated AMTs from *C. reinhardtii*, *Arabidopsis*, rice, *P. patens*, and *C. vulgaris*. The phylogenetic tree revealed two major clades: the AMT1 and AMT2 subfamily (Figure 1). Remarkably, all CrAMT genes were classified within the AMT1 subfamily, and the four AMT genes from *C. vulgaris* also fell under the AMT1 subfamily. Evolutionarily, the AMT2 subfamily was identified in *P. patens*, *Arabidopsis*, and rice, suggesting its uniqueness to both lower and higher plant species. Although we have identified the presence of AMT2 subfamily genes in *P. patens*, the origin of these genes remains unclear.

### 3.3. Characteristics of CrAMT Proteins

**Figure 2:**
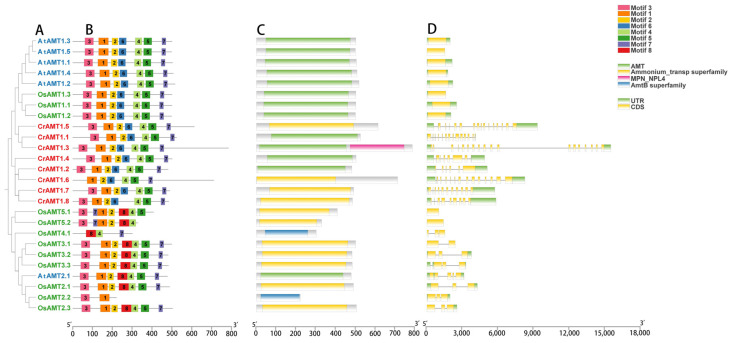
A comparative analysis of CrAMTs, AtAMTs, and OsAMTs. (**A**) A phylogenetic tree highlighting the evolutionary relationships, constructed using MEGA 11 through the maximum-likelihood method. AtAMTs, CrAMTs, and OsAMTs are denoted by blue, red, and green fonts, respectively. (**B**) An analysis of conserved motifs in AMT amino acid sequences conducted via the MEME v5.5.4 online tool, with each color indicating a distinct motif. (**C**) Conserved domain analysis of AMT proteins using the NCBI CD-search tool. (**D**) Gene structure is depicted showing introns and exons, the green and yellow segments represent introns and exons, respectively.

To further analyze the structure of CrAMT proteins, the conserved motifs, domains, and gene structures of CrAMTs, AtAMTs, and OsAMTs were examined in light of their evolutionary relationship (Figure 2). Specifically, most AMT proteins, based on the similarity of protein sequences, exhibited matching homologs and shared conserved motifs and structural domains. Eight conserved motifs were identified using MEMEv5.5.4 (Figure 2B), and a phylogenetic tree was constructed based on their protein sequences, distinguishing the AMT1 and AMT2 subfamilies (Figure 2A). A notable distinction was that CrAMTs possessed motif 7, unique to the AMT1 subfamily, and lacked motif 8, exclusive to the AMT2 subfamily. CrAMT1.6 was devoid of motif 3, suggesting motifs 1, 2, and 4 through 7 as essential for *C. reinhardtii*.

In terms of structural domains, these proteins typically possess an AMT structural domain, an ammonium transporter superfamily domain, or an AMTB superfamily domain, all associated with ammonium transport (Figure 2C). Uniquely, CrAMT1.3 was characterized by an MPN-NPL4 structural domain, indicative of a nuclear protein localization-4 (Npl4) domain. Furthermore, the analysis of gene structures showed that CrAMTs typically possess a significant number of introns, with each gene containing more than six. Notably, CrAMT1.3 had the highest number, with 18 introns (Figure 2D). These results indicate that CrAMTs share similar gene structures, suggesting a high degree of conservation in their gene architecture.

### 3.4. Chromosome Localization Analysis and Collinearity Analysis of CrAMTs

**Figure 3:**
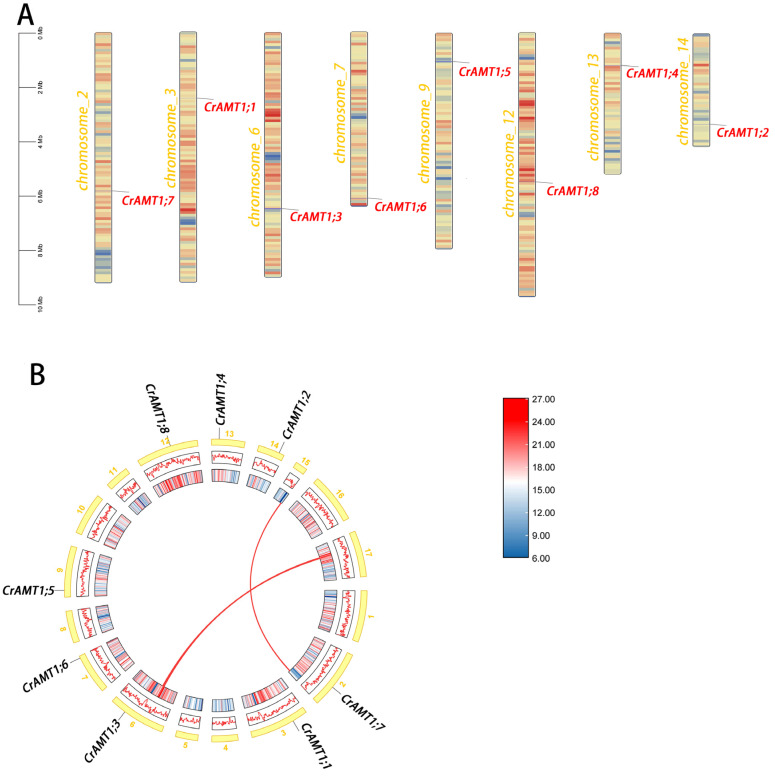
Chromosomal distribution analysis and collinearity analysis of CrAMT genes. (**A**) Chromosomal distribution of the CrAMT genes was visualized using TBtoolsv2.030. Chromosome colors denote *C. reinhardtii* chromosomes, with varying color gradients indicating gene density. Gene names are highlighted in red on their respective chromosomes. A scale bar is provided on the left. Below each chromosome, color gradients illustrate gene density, accompanied by a density scale on the right. (**B**) Genome-wide collinearity analysis, with chromosomes shown in yellow and labeled with their respective numbers. The redlines across chromosomes depict collinear gene pairs within the genome.

Chromosome localization of CrAMT genes was performed using TBtools v2.030 (Figure 3A). The analysis revealed that the CrAMT genes were distributed across various chromosomes. A genome-wide collinearity analysis showed a limited presence of collinear gene pairs, identifying only two such pairs (Figure 3B). Interestingly, none of the CrAMT genes were found within these collinear gene pairs. Gene duplication is a crucial factor in the evolution of organisms [62], and it primarily occurs through two mechanisms: segmental duplication and tandem duplication [62,63]. Considering that the CrAMT genes were localized on different chromosomes and lacked collinearity, it was inferred that their duplication occurs through segmental replication.

### 3.5. Promoter Cis-Element Analysis of CrAMTs

**Figure 4:**
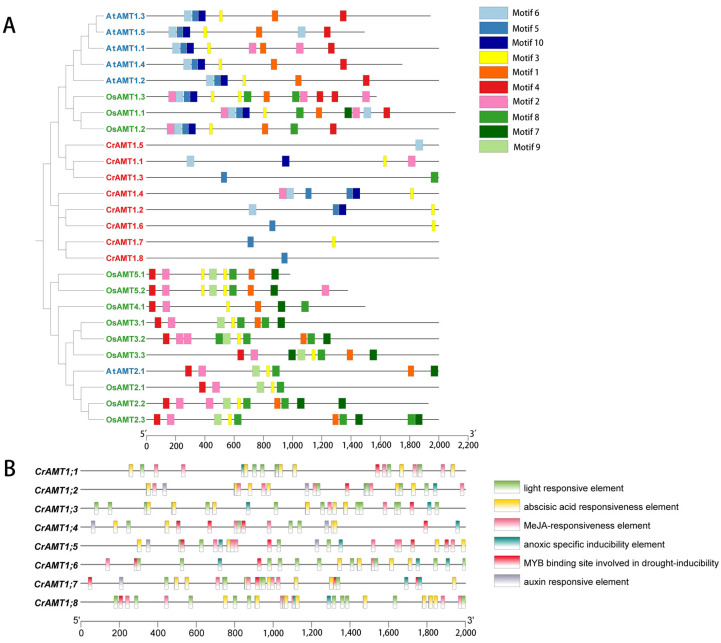
Analysis of promoter *cis*-elements in CrAMTs. (**A**) depicts a phylogenetic tree constructed using MEGA 11, where AtAMTs, CrAMTs, and OsAMTs are indicated in blue, red, and green fonts, respectively. Adjacent to the tree, conserved motifs identified in the promoters of AtAMTs, CrAMTs, and OsAMTs through MEME v5.5.4 analysis are shown, with each color symbolizing a distinct motif. (**B**) showcases the *cis-*element analysis within the promoter regions, 2 kb upstream of CrAMTs, performed via PlantCARE. Various color boxes denote different *cis*-elements, each associated with specific functions as elucidated.

The promoters of CrAMTs, AtAMTs, and OsAMTs were examined for *cis*-elements to understand transcription initiation and regulation. The 2 kb promoter regions upstream of these genes were analyzed for conserved motifs and their evolutionary significance (Figure 4A). The analysis identified distinct differences in promoter sequences among these species. Specifically, motifs 5, 6, and 10 were unique to the AMT1 subfamily, whereas motifs 7, 8, and 9 were predominantly found in the AMT2 subfamily genes. CrAMTs displayed fewer motifs, ranging from one to six, and possessed motifs 5, 6, and 10, characteristic of the AMT1 subfamily.

Promoters include a variety of short *cis*-acting regulatory elements that are crucial for assembling the transcriptional machinery and regulating expression levels and their associated functions [29]. Using TBtools v2.030 and the PlantCare website, we analyzed the 2 kb sequences upstream of the start codon for CrAMTs (Figure 4B). The analysis of the promoter *cis*-element across all CrAMTs revealed a variety of *cis*-elements associated with plant hormone responses and light sensitivity in the promoter region. Light-responsive elements were predominant, underscoring the link between nitrogen utilization and photosynthetic carbon assimilation, crucial for maintaining the carbon–nitrogen balance. Among the hormone-responsive elements, methyl jasmonate (MeJA) response elements were most common, followed by abscisic acid elements present in all genes. Interestingly, auxin response elements were missing in *CrAMT1;1*, *CrAMT1;6*, and *CrAMT1;8*. Stress-related elements, including hypoxia-specific and drought-inducible elements, were identified in all genes.

### 3.6. Expression Patterns of CrAMT Genes under High-Ammonium Conditions

**Figure 5:**
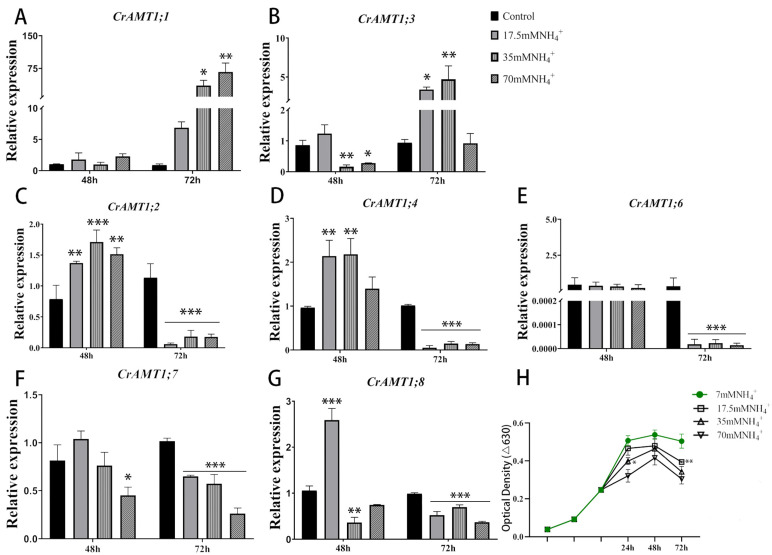
The expression patterns of CrAMT genes and growth trends under high-ammonium conditions. (**A**–**G**) The relative expression level of *CrAMT1;1*, *CrAMT1;3*, *CrAMT1;2*, *CrAMT1;4*, *CrAMT1;6*, *CrAMT1;7*, and *CrAMT1;8* under different ammonium treatments (7 mM, 17.5 mM, 35 mM, 70 mM NH_4_^+^, respectively). The x-axis numbers represent 48 h and 72 h after ammonium treatment of CC-125, respectively. (**H**) depicts the optical density at 630 nm for CC-125 grown under different ammonium concentrations (7 mM, 17.5 mM, 35 mM, 70 mM NH_4_^+^). The x-axis represents 24 h, 48 h, and 72 h after ammonium treatment in the logarithmic phase of growth. The y-axis represents the optical density at 630 nm. The green line indicates growth under normal ammonium conditions (7 mM NH_4_^+^). Legend details are provided on the right. The above data were analyzed using one-way ANOVA followed by Tukey’s test at p < 0.05 with normal culture as control (*p* < 0.05 indicated by *, *p* < 0.01 indicated by **, *p* < 0.001 indicated by ***).

The functional validation of CrAMT genes was conducted by analyzing expression changes through qRT-PCR under various NH_4_^+^ concentrations. Remarkably, *CrAMT1;5′*s expression was undetectable across all tested conditions (Figure 5A–G). The optical density curve of CC-125 at 630 nm indicates that growth was significantly inhibited under NH_4_^+^ treatment (17.5 mM, 35 mM, 70 mM NH_4_^+^) compared to normal TAP medium culture (7 mM NH_4_^+^), with the degree of inhibition escalating with increasing NH_4_^+^ concentrations (Figure 5H). This growth inhibition reflects the toxic impact of excessive NH_4_^+^ on CC-125.

After 72 h of NH_4_^+^ exposure, optical density measurements suggested a significant reduction in growth compared to the 48 h interval, indicating an intensified inhibition effect due to prolonged NH_4_^+^ toxicity. Relative to the 48 h treatment, qRT-PCR analysis at 72 h showed a significant upregulation of *CrAMT1;1* and *CrAMT1;3*, whereas *CrAMT1;2*, *CrAMT1;4*, and *CrAMT1;6* experienced substantial downregulation. *CrAMT1;7* and *CrAMT1;8* expressions also decreased, but less so. Consequently, it is hypothesized that CrAMT genes play a role in NH_4_^+^ uptake and transport. CC-125 likely modulates the absorption and effluxes of NH_4_^+^ in algal cells by regulating the expression of CrAMT genes, thereby adapting to a high-ammonium environment.

### 3.7. Identification and Analysis of CrAMT1;7-OE

Under normal culture conditions, the relative expression levels of all CrAMT genes in CC-125 were assessed. Among them, *CrAMT1;5* was not detected, while *CrAMT1;7* exhibited the highest relative expression (Figure S1). Given the closer proximity of *CrAMT1;7* and *CrAMT1;8* in the phylogenetic tree and their similar expression patterns under high-ammonium treatment, it is hypothesized that *CrAMT1;7* and *CrAMT1;8* may be functionally linked. Therefore, it is suggested to conduct overexpression and knockdown experiments on *CrAMT1;7* and *CrAMT1;8* in algas to further investigate their functional relationship.

**Figure 6:**
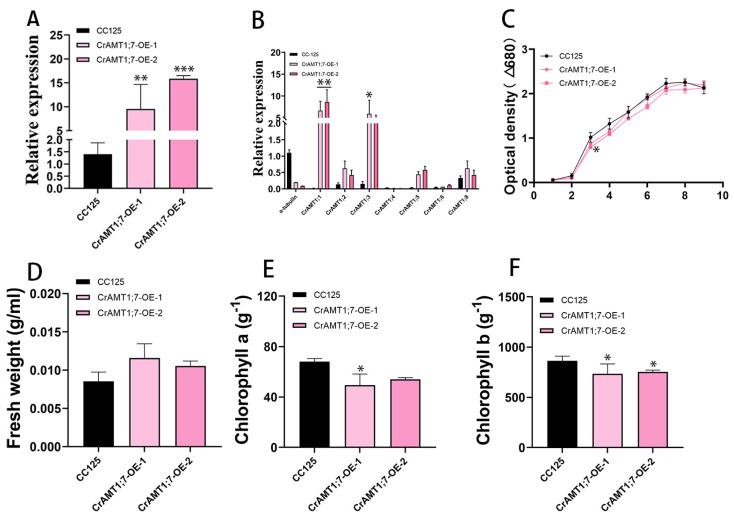
The identification and analysis of CrAMT1;7-OE. (**A**) qRT-PCR analysis showing *CrAMT1;7*’s relative expression of CrAMT1;7-OE, with the x-axis representing different algas and the y-axis indicating relative expression levels. CC-125 is shown as a black bar, while CrAMT1;7-OE is depicted as pink bars (the same below). (**B**) The relative expression levels of other CrAMT genes in CC-125 and CrAMT1;7-OE; we selected a-tubulin from CC-125 as the reference. (**C**) The growth curves of CC-125 and CrAMT1;7-OE in TAP medium, measured according to the optical density at 680 nm every 24 h. The x-axis shows the number of days, with the black line for CC-125 and various pink lines for CrAMT1;7-OE. (**D**) The fresh weight per unit volume at the growth plateau. (**E**) Chlorophyll a content per unit mass at the plateau stage. (**F**) Chlorophyll b content per unit mass at the plateau stage. The above data were analyzed using one-way ANOVA followed by Tukey’s test at p < 0.05 with CC-125 as control (*p* < 0.05 indicated by *, *p* < 0.01 indicated by **, *p* < 0.001 indicated by ***).

Only two transgenic algas were identified, named CrAMT1;7-OE-1 and CrAMT1;7-OE-2. qRT-PCR analysis showed that the relative expression of *CrAMT1;7* increased from approximately 10% to 16% (Figure 6A). The relative expression level of *CrAMT1;1* and *CrAMT1;3* was significantly increased in the qRT-PCR analysis of CC-125 and CrAMT1;7-OE, while the relative expression levels of other genes did not show significance changes (Figure 6B). The similar growth patterns and physiological characteristics of CrAMT1;7-OE and CC-125 at the plateau stage indicate that the overexpression of *CrAMT1;7* may not have a significant impact on these parameters under normal growth conditions (Figure 6C–F).

### 3.8. Identification and Analysis of CrAMT1;7-KD

**Figure 7:**
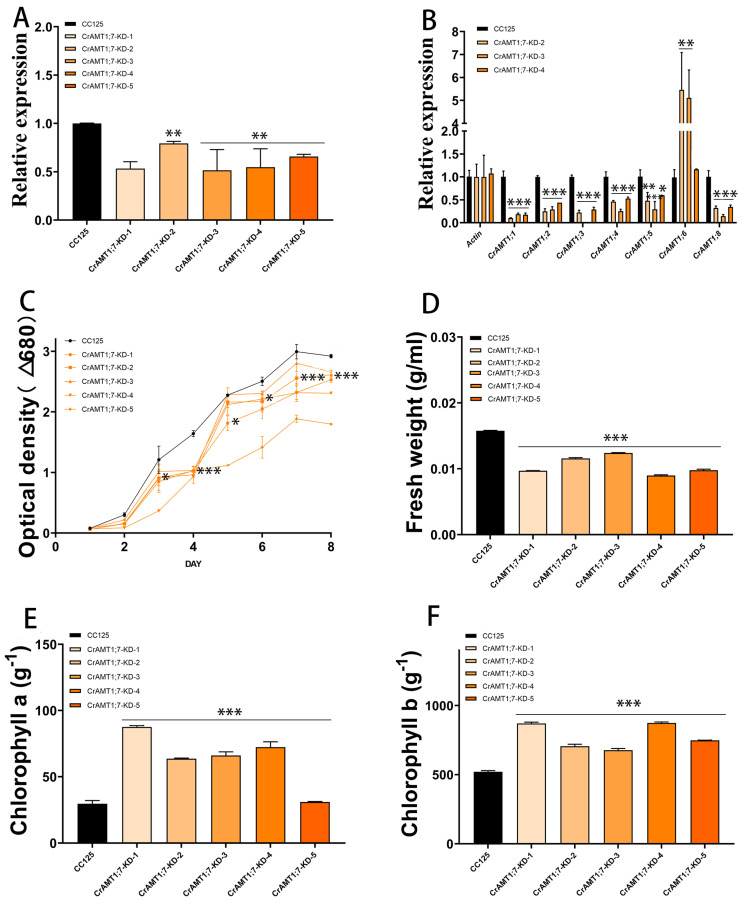
The identification and analysis of CrAMT1;7-KD. (**A**) qRT-PCR analysis illustrating the relative expression levels of *CrAMT1;7* across 5 transgenic algas, with the x-axis indicating different algas and the y-axis showing relative expression levels. The black bar denotes CC-125, and the orange bars represent CrAMT1;7-KD-1 to CrAMT1;8-KD-5 (the same below). (**B**) A comparison of other CrAMT genes’ relative expression in CC-125 and CrAMT1;7-KD-2, CrAMT1;7-KD-3, and CrAMT1;7-KD-4 using qRT-PCR, with Actin from CC-125 as the reference. (**C**) The growth curves of both CrAMT1;7-KD and CC-125 in normal TAP medium, measured according to the optical density at 680 nm every 24 h. The x-axis represents the number of days, with the black line for CC-125 and various orange lines for CrAMT1;7-KD. (**D**) The fresh weight per unit volume at the growth plateau stage. (**E**) Chlorophyll a content per unit mass at the plateau stage. (**F**) Chlorophyll b content per unit mass at the plateau stage for CC-125 and CrAMT1;7-KD. The above data were analyzed using one-way ANOVA followed by Tukey’s test at p < 0.05 with CC-125 as control (*p* < 0.05 indicated by *, *p* < 0.01 indicated by **, *p* < 0.001 indicated by ***).

Five transgenic algas were identified, named CrAMT1;7-KD-1 to CrAMT1;7-KD-5. qRT-PCR analysis indicated a 30% to 40% decrease in the relative expression of *CrAMT1;7* in the transgenic algas compared to CC-125 (Figure 7A). Comparative qRT-PCR analysis of CrAMT1;7-KD-2, CrAMT1;7-KD-3, and CrAMT1;7-KD-4 showed a significant increase in the relative expression of *CrAMT1;6*, while the relative expression of other genes decreased notably (Figure 7B). Growth analysis in normal TAP medium revealed differences between CrAMT1;7-KD and CC-125. The growth trend of CrAMT1;7-KD was consistently lower than that of CC-125, with some slight growth observed in CrAMT1;7-KD after the fourth day, but still below that of CC-125. The optical density of CrAMT1;7-KD at the plateau stage was significantly lower than that of CC-125 (Figure 7C).

The fresh weight per unit volume at the plateau stage for CrAMT1;7-KD was notably lower than that of CC-125 (Figure 7D). Chlorophyll a content per unit mass in CrAMT1;7-KD at the plateau stage was higher than in CC-125, except for CrAMT1;7-KD-5 (Figure 7E). Similarly, chlorophyll b content per unit mass was higher in CrAMT1;7-KD than in CC-125 (Figure 7F).

### 3.9. Identification and Analysis of CrAMT1;8-OE

**Figure 8:**
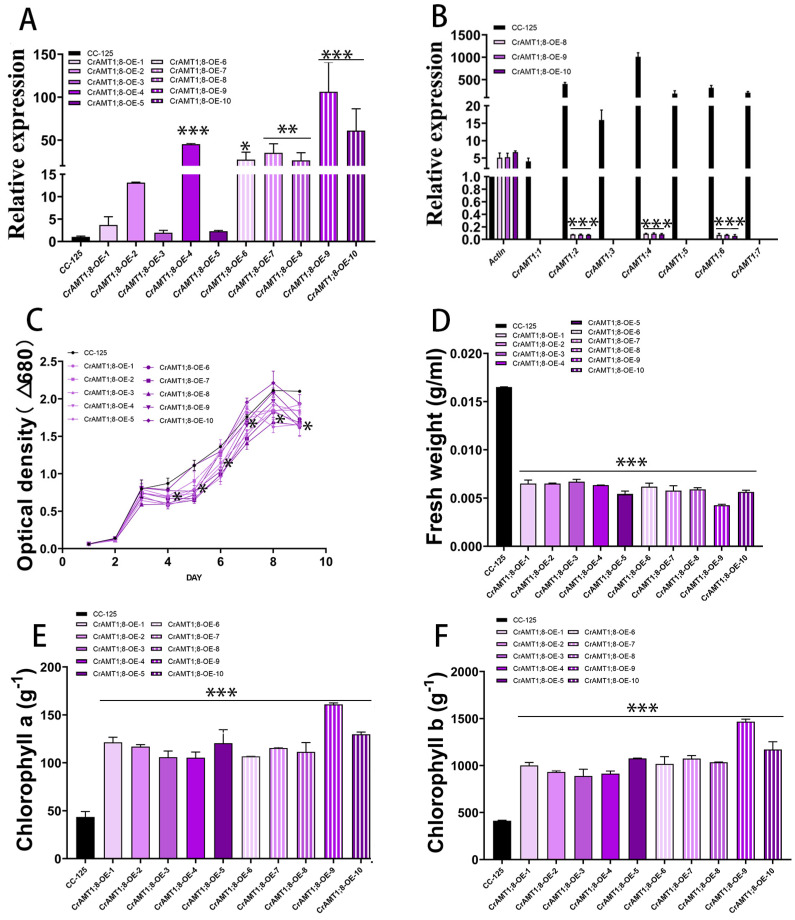
The identification and analysis of CrAMT1;8-OE. (**A**) qRT-PCR analysis showing *CrAMT1;8* relative expression across 10 transgenic algas, with the x-axis representing different algas and the y-axis indicating relative expression levels. CC-125 is shown as a black bar, while CrAMT1;8-OE-1 to CrAMT1;8-OE-10 are depicted as purple bars with varied patterns (the same below). (**B**) The relative expression levels of other CrAMT genes in CC-125 and CrAMT1;8-OE-8, CrAMT1;8-OE-9, and CrAMT1;8-OE-10, with Actin from CC-125 as the reference. (**C**) The growth curves of CC-125 and CrAMT1;8-OE in TAP medium, measured according to the optical density at 680 nm every 24 h. The x-axis shows the number of days, with the black line for CC-125 and various purple lines for CrAMT1;8-OE. (**D**) The fresh weight per unit volume at the growth plateau stage. (**E**) Chlorophyll a content per unit mass at the plateau stage. (**F**) Chlorophyll b content per unit mass at the plateau stage. The above data were analyzed using one-way ANOVA followed by Tukey’s test at p < 0.05 with CC-125 as control (*p* < 0.05 indicated by *, *p* < 0.01 indicated by **, *p* < 0.001 indicated by ***).

Ten transgenic algas were identified, named CrAMT1;8-OE-1 through CrAMT1;8-OE-10. qRT-PCR analysis revealed that CrAMT1;8-OE-4, CrAMT1;8-OE-6, CrAMT1;8-OE-7, CrAMT1;8-OE-8, CrAMT1;8-OE-9, and CrAMT1;8-OE-10 exhibited significant increases in *CrAMT1;8* relative expression, with CrAMT1;8-OE-9 showing the highest level (Figure 8A). A comparison of CC-125 with CrAMT1;8-OE-8, CrAMT1;8-OE-9, and CrAMT1;8-OE-10 indicated a significant reduction in the relative expression of other CrAMTs, with *CrAMT1;1*, *CrAMT1;3*, *CrAMT1;5,* and *CrAMT1;7* almost completely suppressed, and *CrAMT1;2*, *CrAMT1;4,* and *CrAMT1;6* significantly decreased (Figure 8B). Growth analysis of CrAMT1;8-OE and CC-125 in normal TAP medium showed initial similarity in growth trends for the first three days. However, partial CrAMT1;8-OE growth notably slowed on the fourth day, followed by a period of slow growth for 24 h, and then a “J-shaped” growth curve until reaching a plateau. The optical density at 680 nm of partial CrAMT1;8-OE growth at the plateau stage was significantly lower than that of CC-125 (Figure 8C).

The fresh weight per unit volume of CrAMT1;8-OE at the plateau stage was also significantly lower than that of CC-125, with CrAMT1;8-OE-9 having the lowest fresh weight (Figure 8D). Conversely, chlorophyll a content per unit mass in CrAMT1;8-OE at the plateau stage was significantly higher than in CC-125, with CrAMT1;8-OE-9 showing the highest chlorophyll a content (Figure 8E). Similarly, chlorophyll b content per unit mass was significantly higher in CrAMT1;8-OE than in CC-125 during the plateau phase, with CrAMT1;8-OE-9 exhibiting the highest chlorophyll b content (Figure 8F).

### 3.10. Identification and Analysis of CrAMT1;8-KD

**Figure 9:**
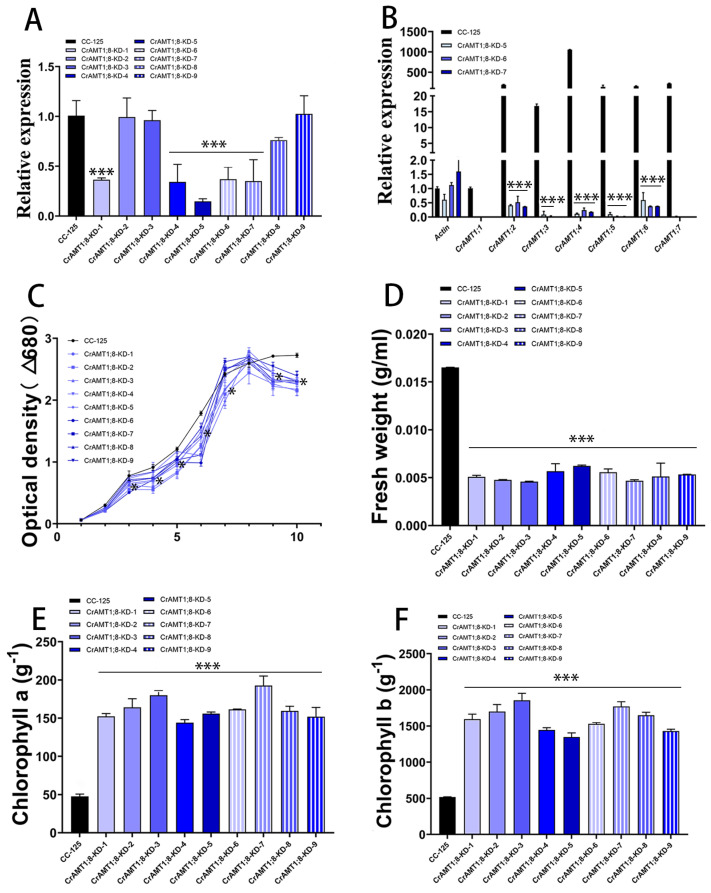
The identification and analysis of CrAMT1;8-KD. (**A**) qRT-PCR analysis illustrating the relative expression levels of *CrAMT1;8* across 9 transgenic algas, with the x-axis indicating different algas and the y-axis showing relative expression levels. The black bar denotes CC-125, and the blue bars represent CrAMT1;8-KD-1 to CrAMT1;8-KD-9 (the same below). (**B**) A comparison of other CrAMT genes’ relative expression in CC-125 and CrAMT1;8-KD-5, CrAMT1;8-KD-6, and CrAMT1;8-KD-7 using qRT-PCR, with Actin from CC-125 as the reference. (**C**) The growth curves of both CrAMT1;8-KD and CC-125 in normal TAP medium, measured according to the optical density at 680 nm every 24 h. The x-axis represents the number of days, with the black line for CC-125 and various blue lines for CrAMT1;8-KD. (**D**) The fresh weight per unit volume at the growth plateau stage. (**E**) Chlorophyll a content per unit mass at the plateau stage. (**F**) Chlorophyll b content per unit mass at the plateau stage for CC-125 and CrAMT1;8-KD. The above data were analyzed using one-way ANOVA followed by Tukey’s test at p < 0.05 with CC-125 as control (*p* < 0.05 indicated by *, *p* < 0.001 indicated by ***).

Nine transgenic algas were identified, named CrAMT1;8-KD-1 to CrAMT1;8-KD-9. qRT-PCR analysis showed a significant reduction in *CrAMT1;8* relative expression in CrAMT1;8-KD-1, CrAMT1;8-KD-4, CrAMT1;8-KD-5, CrAMT1;8-KD-6, and CrAMT1;8-KD-7 (Figure 9A). Comparative qRT-PCR analysis of CC-125 and CrAMT1;8-KD-5, CrAMT1;8-KD-6, and CrAMT1;8-KD-7 revealed near elimination of *CrAMT1;1*, *CrAMT1;3*, *CrAMT1;5*, and *CrAMT1;7* relative expression, as well as significant reductions in *CrAMT1;2*, *CrAMT1;4*, and *CrAMT1;6* relative expression (Figure 9B). Growth analysis in normal TAP medium of CrAMT1;8-KD alongside CC-125, with the optical density at 680 nm measured every 24 h, indicated that the growth patterns of CrAMT1;8-KD initially mirrored those of CC-125 for the first 3 days. However, starting from the third day, the growth of some CrAMT1;8-KD was significantly lower than that of CC-125, and by the time they reached the plateau stage, the optical density of all CrAMT1;8-KD was significantly lower than that of CC-125 (Figure 9C).

The fresh weight per unit volume at the plateau stage for CrAMT1;8-KD was notably lower than for CC-125 (Figure 9D). Chlorophyll a content per unit mass in CrAMT1;8-KD at the plateau stage was higher than in CC-125 (Figure 9E). Similarly, chlorophyll b content per unit mass was higher in CrAMT1;8-KD than in CC-125 (Figure 9F).

### 3.11. Growth Trend of Transgenic algas under High-Ammonium Treatment

**Figure 10:**
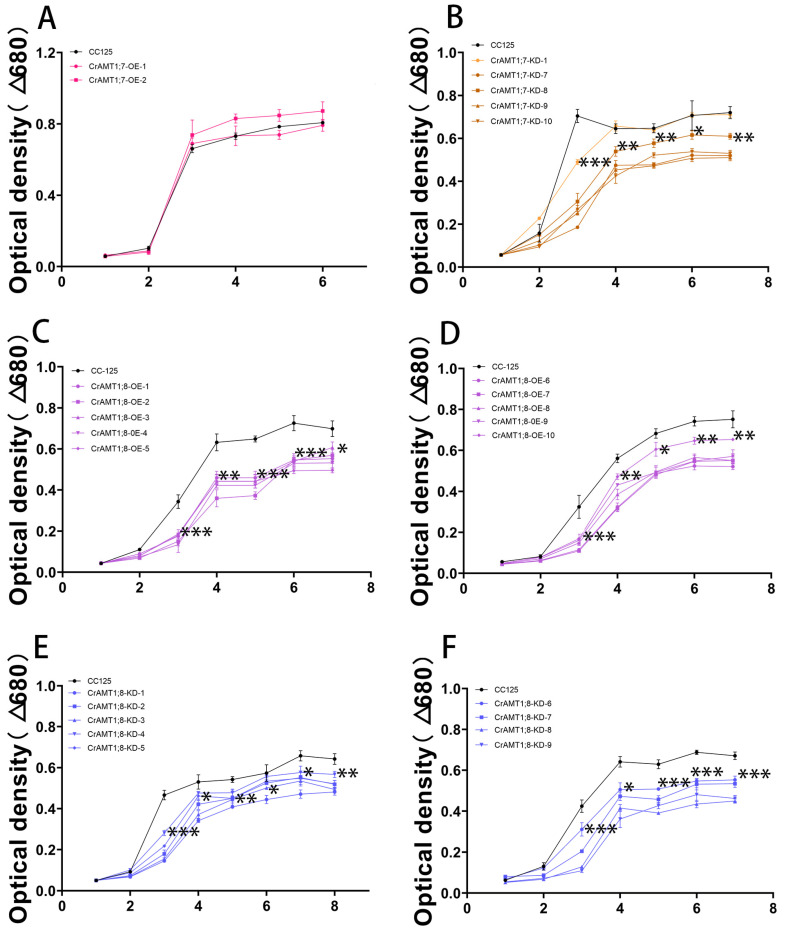
Growth trend of transgenic algas under high-ammonium treatment. (**A**) Growth trend of CrAMT1;7-OE under 35 mM NH_4_^+^. (**B**) Growth trend of CrAMT1;7-KD. (**C**,**D**) Growth trend of CrAMT1;8-OE. (**E**,**F**) Growth trend of CrAMT1;8-KD. The above data were analyzed using one-way ANOVA followed by Tukey’s test at p < 0.05 with CC-125 as control (*p* < 0.05 indicated by *, *p* < 0.01 indicated by **, *p* < 0.001 indicated by ***).

Treating CrAMT1;7-OE, CrAMT1;7-KD, CrAMT1;8-OE, and CrAMT1;8-KD with 35 mM NH_4_^+^ as toxic conditions, the absorbance values of the algas at 680 nm were recorded every 24 h. Overall, the growth trends of CrAMT1;7-KD, CrAMT1;8-OE, and CrAMT1;8-KD were significantly lower than that of CC-125, except for CrAMT1;7-KD-1, which showed no significant difference in growth trend compared to CC-125 after the fourth day (Figure 10B–F). Meanwhile, CrAMT1;7-OE under high-ammonium treatment exhibited no significant difference in growth trend compared to CC-125 (Figure 10A).

## 4. Discussion

The AMT gene family has been extensively studied and identified in various plants such as rice, wheat, and maize [64,65,66]. However, knowledge about the AMT gene family in ammonium-preferring lower species like *C. reinhardtii* is still limited. In this study, we conducted a comprehensive genome analysis of *C. reinhardtii* and identified eight CrAMT genes (Figure 1). AMTs can be classified into two subfamilies: AMT1 and AMT2 [21]. Phylogenetic analysis revealed that both AMT1 and AMT2 subfamily members were present in *P. patens*, *Arabidopsis*, and rice. Notably, all CrAMTs were found to be members of the AMT1 subfamily, similar to those in *C. vulgaris* (Figure 1), suggesting a progressive divergence in the AMT gene family during evolution. This evolution likely resulted in sequence and structural differences between the subfamilies [23,24,25], giving rise to the AMT2 subfamily. However, it remains unclear whether AMT2 subfamily members existed before mosses. Furthermore, there were significant differences in the gene structures of CrAMTs compared to OsAMTs and AtAMTs, with CrAMTs having longer gene lengths and more introns. For instance, *CrAMT1;3* contained 18 introns (Figure 2D). It is generally believed that the number of introns is closely related to the complexity of eukaryotic genomes, with more complex organisms having a greater number of introns [67,68]. In contrast, higher plants such as rice and *Arabidopsis* lack introns in their AMT1 genes. Conservation motif analysis also revealed that AMT1 genes in CrAMTs, AtAMTs, and OsAMTs all share motifs 1 through 7 (Figure 2B). It is speculated that AMT1 genes from AtAMTs, and OsAMTs may have originated from retrotranscription. Additionally, we analyzed the collinearity between *C. reinhardtii* and other species such as *P. patens*, *Arabidopsis*, and rice, and found no collinearity information. This supports the hypothesis that intronless AMT1 genes could have formed. Promoter regions, which drive gene expression, contain *cis*-elements that determine the activity of genes [67]. Surprisingly, despite belonging to the AMT1 subfamily, CrAMTs have fewer conserved motifs in their promoter regions compared to OsAMTs and AtAMTs, which may also relate to the evolution of intronless AMT1 genes. Additionally, we discovered that the promoter regions of CrAMTs include light-responsive *cis*-elements, likely reflecting the interplay between nitrogen uptake and carbon assimilation necessary to maintain the carbon–nitrogen balance in *C. reinhardtii*.

Physicochemical experiments have shown that the relative expression of *CrAMT1;1* and *CrAMT1;3* significantly increased under NH_4_^+^ treatment, whereas the relative expression of *CrAMT1;2*, *CrAMT1;4*, and *CrAMT1;6* decreased notably. Additionally, *CrAMT1;7* and *CrAMT1;8* exhibited a general trend of downregulation (Figure 5). This pattern aligns with the division of CrAMTs into three branches on the phylogenetic tree.

Although *CrAMT1;7* exhibited the highest relative expression level among all CrAMT genes under normal conditions, neither overexpression nor knockdown resulted in improved growth compared to CC-125. In CrAMT1;7-OE, the relative expression levels of *CrAMT1;1* and *CrAMT1;3* significantly increased, while in CrAMT1;7-KD, they decreased notably. These two genes not only clustered closely together in the phylogenetic tree but also showed a significant increase in relative expression after 72 h of high-ammonium treatment. This indicates that CrAMT1.1, CrAMT1.3, and CrAMT1.7 are functionally related. We speculate that this may be attributed to CrAMT1.7 being involved in the transport of NH_4_^+^ into algal cells, while CrAMT1.1 and CrAMT1.3 are involved in transporting NH_4_^+^ from algal cells to be stored in vacuoles.

Both CrAMT1;8-OE and CrAMT1;8-KD algas exhibited lower growth rates and fresh weight per unit volume compared to CC-125, accompanied by suppressed or absent expression in other CrAMT genes. This underscores the critical role of the *CrAMT1;8* gene in maintaining the stability of *C. reinhardtii*, as disruptions in this gene significantly affected the relative expression levels of other CrAMT genes and consequently inhibited growth. Intriguingly, both the CrAMT1;8-OE and CrAMT1;8-KD algas showed a significant increase in chlorophyll content per unit mass compared to CC-125. This observation suggests a potential link between *CrAMT1;8* and chlorophyll synthesis, though the specific molecular mechanisms remain to be elucidated. Understanding these regulatory pathways can reveal how algal cells adapt to changes in their environment, particularly in response to variations in nutrient availability.

In conclusion, this study has laid a foundational understanding of CrAMT genes and their protein expressions in *C. reinhardtii*. We explored the trends in gene expression under different ammonium treatment conditions and examined the specific roles of *CrAMT1;7* and *CrAMT1;8*. Clearly, CrAMTs play a crucial role in ammonium uptake and transport, with each gene carrying different functional responsibilities that interact with each other. The stability of *CrAMT1;7* and *CrAMT1;8* is essential for the overall function of CrAMTs. While we expected transgenic algas with increased tolerance to high ammonium environments, the overexpression or knockdown of *CrAMT1;7* and *CrAMT1;8* did not exhibit the anticipated enhanced tolerance phenotypes. This could be attributed to the growth status being inherently suppressed in CrAMT1;7-KD, CrAMT1;8-OE, and CrAMT1;8-KD, thereby rendering them unable to withstand external high-ammonium environments. Further research is crucial for elucidating the molecular mechanisms behind the perception, uptake, and transport of ammonium in *C. reinhardtii.* Studies have demonstrated that ammonium accelerates damage to photosystem II in Synechocystis sp. PCC6803. Disrupting psbA1, which encodes the D1 protein of photosystem II, leads to heightened sensitivity to ammonium toxicity [49,69]. Expanding on this understanding, we can develop mitigation strategies or continue investigating beneficial CrAMT genes. It is noteworthy that CrAMTs do not encompass members of the AMT2 subfamily. Hence, hypotheses can be formulated concerning the role of exogenous AMT2 genes in *C. reinhardtii*.

## 5. Conclusions

In this study, we performed a thorough analysis of the *C. reinhardtii* genome and successfully identified eight members of the CrAMT family, all classified within the AMT1 subfamily. These genes are characterized by a uniform structure with a high number of introns, are distributed across eight chromosomes without any tandem repeats, exhibit conserved motifs typical of the AMT protein family, and have promoter regions which consistently contain light-responsive and plant hormone-responsive *cis*-elements. CrAMT genes display three distinct expression patterns under ammonium concentrations, corresponding to the three branches of the phylogenetic tree. Our transgenic experimental results highlight that the maintenance of *CrAMT1;7* and *CrAMT1;8* is critical for *C. reinhardtii* and that their precise mechanisms of ammonium transport need to be further elucidated through molecular and genetic studies. Although the overexpression or knockdown of *CrAMT1;7* and *CrAMT1;8* did not demonstrate the expected enhanced ammonium tolerance phenotypes, these insights provide valuable reference data for further exploration of the functions of CrAMT genes and the development of ammonium-tolerant algas.

## Figures and Tables

**Table 1:** Sequences of qRT-PCR primers.

Gene Name	Primer Sequence (5 ‘→3’)
Actin	F:CAGTAGGAGGCATAGGGTTTGG
	R:TCAACGAATTGGGGTGTGTG
a-tubulin	F:TGCTGTGGGACCTGGCTGA
	R:GCCTTCTTGCTGGTGATGTTG
*CrAMT1;1*	F:ACGGTGCCTACGGATTCGGAAT
	R:AGCCCAGCCTTGTTGAGGATGA
*CrAMT1;2*	F:GCCACACATTCACGCATCCCA
	R:CGTCCAGCCTTCTCGCAATCAG
*CrAMT1;3*	F:AGCTGAGCCTCTGTCACATGGT
	R:GGACGCAGCAAGTCAACCGATT
*CrAMT1;4*	F:GCCGACATGACAACGCACATTG
	R:GCCACGCCCTTTCTCCAAGATG
*CrAMT1;5*	F:CATCCAGTACGGCGATCCTCCA
	R:TGACACCCACCCTAAGCAGCAA
*CrAMT1;6*	F:CGGTGCTGCCTCGACATAAGTG
	R:GCTCCGCTTGCTGTCATCTCTG
*CrAMT1;7*	F:CGCTGCTGGTTGATGCCTGAA
	R:GCACGCACTGCTTCCACTCTAT
*CrAMT1;8*	F:GTGTGAGGTGGGCGTGTTCAAA
	R:TGCCAGCGAAGACCGATAACCA

**Table 2:** Physicochemical properties of CrAMT proteins.

Gene Name	Protein Name	Gene locus	No. of Amino Acids (a.a.)	Length of CDS (bp)	Transmembrane Zone	Subcellular Localization	
						Plasma Membrane	Vacuolar Membrane
*CrAMT1;1*	CrAMT1.1	Cre03.g159254	521	1566	11	12	1
*CrAMT1;2*	CrAMT1.2	Cre14.g629920	478	1437	10	9	3
*CrAMT1;3*	CrAMT1.3	Cre06.g293051	782	2349	11	11	2
*CrAMT1;4*	CrAMT1.4	Cre13.g569850	499	1500	11	10	2
*CrAMT1;5*	CrAMT1.5	Cre09.g400750	610	1833	10	10	1
*CrAMT1;6*	CrAMT1.6	Cre07.g355650	708	2127	11	7	6
*CrAMT1;7*	CrAMT1.7	Cre02.g111050	487	1464	11	9	1
*CrAMT1;8*	CrAMT1.8	Cre12.g531000	481	1446	10	4	4

## Data Availability

The cited sequence data can be found in the EMBL/GenBank data libraries under accession number(s): *AtAMT1;1* NP_193087.1, *AtAMT1;2* AEE34288.1, *AtAMT1;3* AEE76886.1, *AtAMT1;*4 Q9SVT8.1, *AtAMT1;5* Q9LK16.1, *AtAMT2;1* ABF57277.1, *OsAMT2;3* NP_915337.1, *OsAMT3;1* BAC65232.1, *OsAMT3;2* AAO41130, *OsAMT3;3* Q69T29, *OsAMT4;1* Q10CV4, *OsAMT5;1* Os12g01420.1, *OsAMT5;2* Os11g01410.1, Pp3c2_15630 XP_024399300.1, Pp3c12_11680 PNR43772.1, Pp3c16_22550 XP_024397895.1, Pp3c22_5530 XP_024360240.1, Pp3c19_2270 PNR33761.1, Pp3c6_17000 PNR52687.1, Pp3c14_13820 PNR41085.1, Pp3c16_12080 XP_024399507.1, Pp3c25_12520 XP_024365167.1, Pp3c5_13670 XP_024375173.1, Pp3c10_10460 XP_024387479.1, Pp3c18_22220 XP_024401782.1, Pp3c21_10030 XP_024359985.1, Pp3s397_40 XP_024367994.1, Pp3c21_10000 XP_024359294.1, Pp3c18_18430 XP_024403228.1, Pp3s397_10 PNR26033.1, Pp3c18_18460 XP_024402884.1, KAI3431805 KAI3431805.1, KAI3435774 KAI3435774.1, KAI3424104 KAI3424104.1, and KAI3429768 KAI3429768.1.

## Supplementary Materials

The following supporting information can be downloaded at: https://www.mdpi.com/article/10.3390/genes15081002/s1, Figure S1: Expression level of CrAMT genes in plateau stage under normal TAP culture.
