# Supplementary material for: Genome-Wide Identification and Characterization of Ammonium Transporter (AMT) Genes in Chlamydomonas reinhardtii
Source: Genes (Basel). 2024 Jul 31;15(8):1002. doi: 10.3390/genes15081002 (PMC11353525; doi:10.3390/genes15081002)

Figure S1. Expression level of CrAMT genes in plateau stage under normal TAP culture. The x-axis numbers represent relative expression level of *CrAMT1;1*, *CrAMT1;2*, *CrAMT1;3*, *CrAMT1;4*, *CrAMT1;5*, *CrAMT1;6*, *CrAMT1;7*, and *CrAMT1;8*. Actin from CC-125 as the reference.

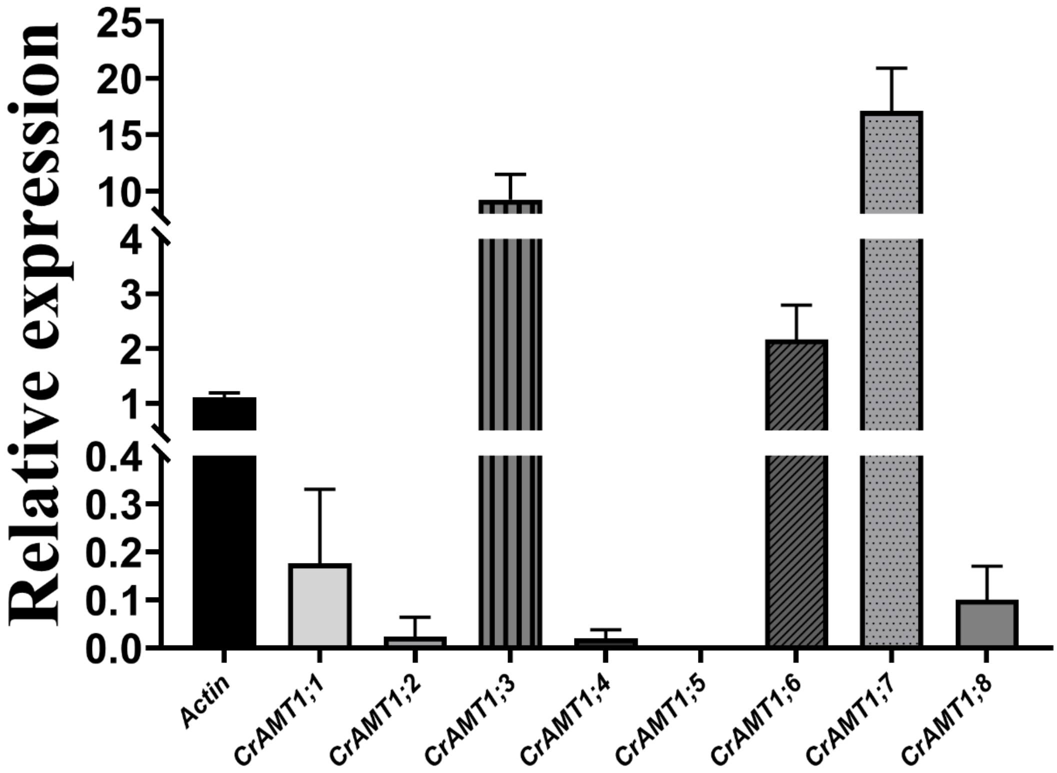

Supplement: Supplementary file 1 [file genes-15-01002-s001.zip › genes-3116362-supplementary.pdf]
